# Integrin **α**4**β**7 as a predictor of HIV acquisition: one thread in a complex tapestry

**DOI:** 10.1172/JCI195258

**Published:** 2025-08-01

**Authors:** Tosin E. Omole, Lyle R. McKinnon

**Affiliations:** 1 Department of Medical Microbiology and Infectious Diseases, University of Manitoba, Winnipeg, Manitoba, Canada.; 2 Vaccine and Infectious Disease Organization (VIDO), Saskatoon, Saskatchewan, Canada.; 3 Centre for the AIDS Programme of Research in South Africa (CAPRISA), Durban, South Africa.; 4 Department of Medical Microbiology and Immunology, University of Nairobi, Nairobi, Kenya.

## Abstract

Cellular susceptibility to HIV is associated with integrin α4β7, a mucosal homing receptor involved with trafficking HIV target cells to sites of HIV replication. However, studies investigating preinfection α4β7 expression as a predictor of HIV outcomes have yielded inconsistent findings, raising questions about the role of α4β7 in HIV acquisition across populations. In this issue of the *JCI*, Machmach et al. assessed PBMCs collected before HIV infection and found higher α4β7 expression on memory CD4^+^ T cells and invariant NK T (iNKT) cells in individuals who went on to acquire HIV. Here, we consider possible explanations that may underlie discrepancies among studies and suggest that α4β7 should be considered as part of a multifactorial profile for determining HIV risk. While unlikely to serve as a target for HIV prevention or therapy, α4β7-directed interventions may offer adjunctive benefits in preserving or improving mucosal immunity.

## T cells expressing the gut-homing integrin α4β7

The odds of HIV transmission are determined by both the infectiousness of the donor and the susceptibility of the recipient. While measuring HIV exposure is feasible in certain contexts such as serodiscordant couples, it remains difficult in many observational studies. Consequently, much of the research on HIV acquisition has focused on identifying biological and epidemiological factors that influence recipient susceptibility. Inflammation and its downstream effects, including recruitment of HIV target cells and compromised mucosal barrier integrity, are considered central to host vulnerability (1, 2). Among HIV’s primary targets are CD4^+^ T cells, which are heterogeneous in their ability to support infection and viral propagation (3). A subset of these cells expressing the gut-homing integrin α4β7 have received considerable attention for their potential role in HIV susceptibility and pathogenesis.

Although a role for α4β7 in HIV cure strategies remains limited (4–6), α4β7-expressing CD4^+^ T cells are consistently characterized as highly permissive to HIV. These cells are among the first to be depleted during acute infection (7), underscoring their preferential targeting. They often coexpress CCR5, the main HIV coreceptor, and markers of Th17 cells, another highly susceptible subset (7, 8). The inherent role of these T cells in trafficking cells to mucosal tissues through interaction with mucosal addressin cell adhesion molecule 1 (MAdCAM-1) enables their strategic localization at anatomical sites critical for HIV transmission and replication, maintaining a pool of target cells for infection during exposure (9). In vitro studies also suggest that some HIV strains can bind α4β7, not as an essential coreceptor, but as an auxiliary adhesion factor that facilitates virus attachment (10, 11). The relationship between preinfection α4β7 expression on memory CD4^+^ T cells and HIV acquisition risk has been investigated across multiple cohorts, with inconsistent findings. As such, α4β7 as a reliable predictor of HIV acquisition in humans remains contentious.

## α4β7 as part of a composite immune signature

In this issue of the *JCI*, Machmach et al. (12) add evidence to the evolving literature on α4β7 and HIV susceptibility. Their study assessed α4β7 expression on PBMCs collected a median of 79 days prior to HIV infection in the RV217 study cohort, which consists of predominantly male and transgender individuals in Thailand at high risk of HIV exposure. The analysis compared 25 highly exposed seroconverters (HESCs) with 74 matched highly exposed seronegative (HESN) controls. Consistent with prior hypotheses, HESC individuals had higher α4β7 expression on memory CD4^+^ T cells and invariant NK T (iNKT) cells. These findings support the idea that elevated α4β7 expression may increase an individual’s susceptibility to HIV acquisition, particularly when expressed on cell subsets already highly permissive to HIV. Interestingly, the study also observed lower α4β7 expression on NK cells in HESC individuals, which suggests a reduced capacity for these cells to home to mucosal sites where early viral control is critical. Affected NK cells showed a decreased response to HIV-infected cells, as indicated by their diminished activity against cells coated with plasma from people with HIV, implying impairment in their antiviral function. Ultimately, α4β7 expression contributed to a composite immune signature, when combined with NK cell activation and iNKT transcriptional markers, that most effectively distinguished HESC from HESN participants. These results suggest that, while α4β7 may lack consistent predictive power on its own, its utility may lie in combination with other immune correlates as part of a multifactorial risk profile. Thus, α4β7 should be viewed not solely as a marker of cellular susceptibility, but as a component of a broader immunological landscape that collectively shapes the risk of HIV acquisition.

The promise of α4β7 as a biomarker of HIV risk was first raised in our earlier work, which showed that higher α4β7 expression on CD4^+^ T cells predicted both increased acquisition and accelerated disease progression among South African women in the CAPRISA 004 trial (13). However, follow-up studies have yielded divergent findings. For example, a multi-cohort study involving men who have sex with men (MSM), transgender individuals, and people who inject drugs (PWIDs) in the United States (HVTN 505 and ALIVE cohorts) found no association and a protective signal in PWIDs (14). In our more recent work examining preinfection PBMCs across African cohorts – HVTN 503 in South Africa and Partners PrEP and COS cohorts in East Africa — α4β7 was not a predictor of HIV acquisition (15). Rather, opposing trends emerged: in South African women especially (HVTN 503), α4β7 expression tended to correlate with greater risk, while in Ugandan participants, higher expression appeared to be protective. Only HVTN 503 showed an association with faster progression (15). These differences suggest region- or context-specific effects that complicate the interpretation of α4β7’s role.

**Figure 1:**
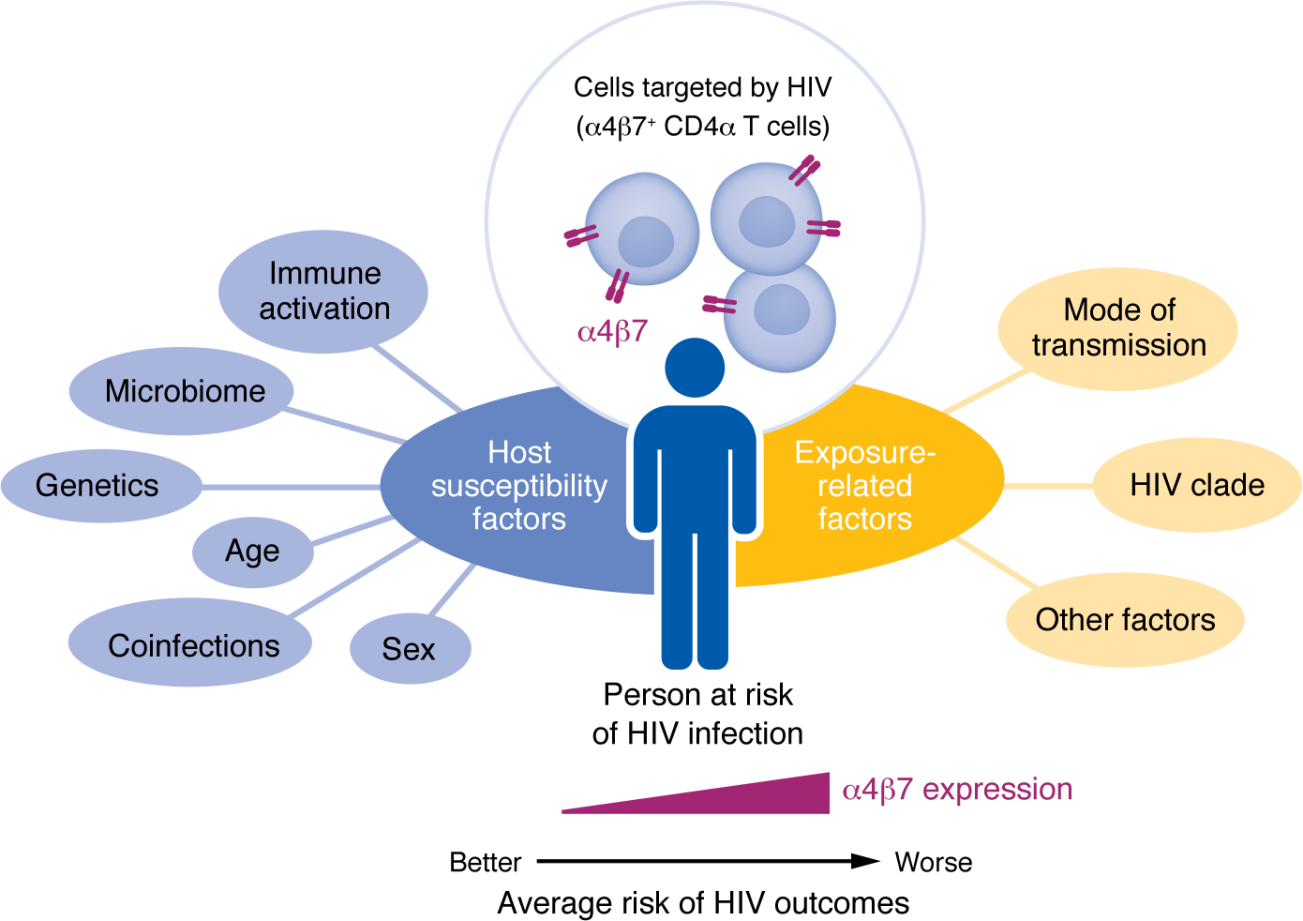
Complex considerations of interacting biological and epidemiological factors that influence the rates of HIV transmission. Host and exposure factors may influence α4β7 frequencies and/or determine associations between α4β7 and HIV outcomes. Interpreting α4β7 in the context of multiple, interacting biological and epidemiological factors is critical when assessing HIV acquisition risk. A similar concept may apply to additional biological predictors or mediators of HIV clinical outcomes.

The recent RV217 study extends this line of research by incorporating α4β7 expression into a multidimensional immune framework (Figure 1) (12). While measuring multiple susceptibility factors in parallel is ideal, Machmach and colleagues also shed more light on the specific HIV target cell hypothesis that has been raised for α4β7 based on a large body of evidence suggesting that α4β7-expressing cells have increased HIV susceptibility (16). Variability in findings between studies may reflect differences in cohort composition, viral clade, transmission route, age distribution, and other factors. For instance, HIV clades AE and C, which are predominant in Thailand and South Africa, respectively, have been shown to bind α4β7 more efficiently in vitro (13, 17), potentially explaining stronger associations in those regions. Likewise, sex-based biological differences in mucosal immunity and route of HIV transmission may affect host associations of HIV risk. Age-related changes in naive and memory T cell subsets and prior exposure history may modulate the relationship between α4β7 and HIV risk; it should be noted that virtually all α4β7^+^ T cells are in the memory compartment, and as individuals age, their naive:memory compartment contracts, affecting α4β7 expression levels. While age was matched and/or adjusted for in most studies, it is noteworthy that stronger α4β7 associations have often been observed in younger cohorts. Additionally, reliance on behavioral criteria to define high-risk groups introduces uncertainty in exposure levels, and small sample sizes may further limit statistical power. Another limitation is the lack of mucosal and tissue sampling in most studies of HIV susceptibility, precluding evaluation of immunological factors at sites of HIV exposure.

## Clinical implications

Beyond its biological plausibility, interest in α4β7 stems from its therapeutic potential. Monoclonal antibodies targeting the α4β7 pathway are approved and considered first-line therapy for the treatment of inflammatory bowel disease (IBD) (18), prompting speculation about their potential use in HIV, where gastrointestinal (GI) disturbances have also been well documented. While it is unlikely these agents will be repurposed as standalone interventions for HIV prevention or cure, they may have adjunctive benefits, such as reducing gut inflammation and restoring mucosal immunity. Supporting this concept, a study in SIV-infected macaques receiving antiretroviral therapy found that combining anti-α4β7 antibodies with IL-21 enhanced immune responses, preserved mucosal T cells, and improved gut microbiome recovery (19). Although the therapy did not prevent viral rebound after antiretroviral therapy (ART) interruption, modest improvements in post-treatment viral control were observed, reinforcing interest in α4β7 as a potential immunomodulatory target. Studies have also suggested that anti-α4β7 monoclonal antibody reduces lymphoid aggregates in the GI tract, which could contribute to its influence on mucosal inflammation (20, 21). Whether anti-α4β7 monoclonal antibody effects in IBD can translate into improved gut health in people living with HIV remains to be seen.

As HIV incidence declines globally, identifying biological predictors of HIV acquisition in prospective cohort studies will become increasingly difficult, underscoring the need for meta-analyses that combine immunologic and exposure data across cohorts. In parallel, continued research into immune signatures of susceptibility and pathogenesis, such as those involving α4β7, can enhance our understanding of host-virus dynamics and help identify vulnerable populations who may benefit most from intensified intervention strategies. Importantly, the emergence of highly effective long-acting preexposure prophylaxis (PrEP) offers a powerful tool that can overcome heterogenous HIV risk that exists within populations (22). While such interventions and general declines in HIV incidence will hamper efforts to study natural HIV acquisition, ensuring equitable access to these biomedical interventions remains a public health imperative that can have a substantial effect on controlling the HIV pandemic.

## Version 1 08/01/2025

Electronic publication
